# Clonidine In Paediatrics – A Review

**Published:** 2009-06

**Authors:** Sujatha Basker, Georgene Singh, Rebecca Jacob

**Affiliations:** 1 Asst. Professor, Department of Anaesthesiology and Critical Care, Christian Medical College, Vellore, Tamil Nadu. 632004; 2 Asst. Professor, Department of Anaesthesiology and Critical Care, Christian Medical College, Vellore, Tamil Nadu. 632004; 3 Professor, Consultant Anaesthesiologist, Bangalore Baptist Hospital, Bangalore, Karnataka. 560024

**Keywords:** Clonidine, Paediatric Anaesthesia, Analgesia, Premedication

## Abstract

**Summary:**

Clonidine, an alpha-2 agonist is a known antihypertensive agent. Because of its sedative and analgesic effects, it is gaining popularity in anaesthesiology. It can be used to premedicate children, as an adjuvant to regional and general anaesthesia and it has several other applications in paediatric anaesthesia. It has also found use in the paediatric intensive care as a sedative, analgesic and to ensure haemodynamic stability. As in the case of any other anaesthetic drug, its use has to be vigilantly monitored.

## Introduction:

Clonidine is a mixed alpha-1 and alpha-2 adrenoceptor agonist with a predominant alpha-2 action. Traditionally, it has been used as an antihypertensive agent since the late sixties. Its primary effect is sympatholysis and it reduces peripheral norepinephrine release by stimulation of the prejunctional inhibitory alpha-2 adrenoceptors. Further uses based on its sedative, anxiolytic and analgesic properties are being developed^1^.

### Pharmacokinetics and Pharmacodynamics:

**Fig 1:**
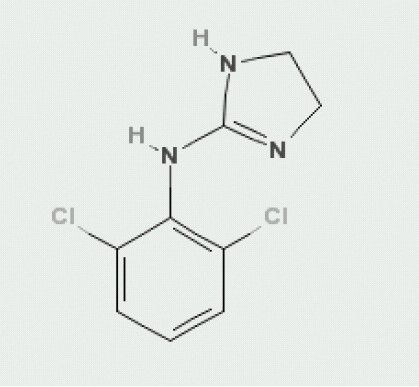
Clonidine(C_9_H_9_Cl_2_N_3_)

Clonidine is N-(2,6 dichlorophenyl)-4,5-dihydro-1H- imidazol-2-amine (Fig 1) with a formula of C_9_H_9_Cl_2_N_3_.

Clonidine is rapidly absorbed after oral administration. It reaches a peak plasma concentration within 60-90 minutes. The bioavailability of the drug is about 75-95%. About 20-40% of the drug is bound to protein. 50% of the drug is metabolized in the liver to inactive metabolites which are excreted in the urine and the half life is about 12-33 hours. As clonidine is lipid soluble, it penetrates the blood-brain harrier to reach the hypothalamus and medulla. It does not require transformation into another substance prior to its action^2^.

Clearance of clonidine in neonates is about one-third of that described in adults due to immature elimination pathways and it reaches about 82% of adult rate by one year of age. Hence maintenance dosing which is a function of clearance should be decreased in neonates and infants when using a target concentration approach^3^.

Rectal administration of 2.5 mcg.kg^−1^ of clonidine in children, approximately 20 minutes before induction of anaesthesia, achieves a plasma concentration within the range known to be clinically effective in adults^4^.

### Mechanism of action:

Alpha-2 adrenergic agonists produce clinical effects by binding to alpha-2 receptors of which there are 3 subtypes: alpha-2a, alpha-2b and alpha-2c. Alpha-2a receptors mediate sedation, analgesia and sympatholysis. Alpha-2b receptors mediate vasoconstriction and possibly anti-shivering mechanisms. The startle response reflects activation of alpha-2c receptors^5^ and it is the response of mind and body to a sudden unexpected stimulus, such as a flash of light, a loud noise (acoustic startle reflex), or a quick movement near the face. In human beings, the reaction includes physical movement away from the stimulus, a contraction of the muscles of the arms and legs, blinking and it also includes blood pressure, respiration, and breathing changes^6^. Clonidine is a centrally acting selective partial adrenergic agonist (alpha-2: alpha-1=220:1).

Alpha-2 receptors are found densely in the pontine locus coeruleus which is an important source of sympathetic nervous system innervation of the forebrain and a vital modulator of vigilance. The sedative effects evoked by alpha-2 agonists most likely reflect inhibition of this nucleus.

Clonidine also stimulates alpha-2 adrenergic inhibitory neurons in the medullary vasomotor centre. As a result, there is a decrease in the sympathetic nervous system outflow from the central nervous system (CNS) to the peripheral tissues. This causes central and peripheral attenuation of sympathetic outflow and central activation of nonadrenergic imidazoline preferring, receptors. Decreased sympathetic nervous system activity is manifested as peripheral vasodilatation and a decrease in systolic blood pressure, heart rate and cardiac output^7^–^9^. The ability of clonidine to modify the potassium channels in the CNS and thereby hyperpolarize the cell membranes may be the mechanism for profound decrease in anaesthetic requirements produced by clonidine.

Neuraxial placement of clonidine inhibits spinal substance P release and nociceptive neuron firing produced by the noxious stimulation. Alpha-2 afferent terminals are situated centrally and peripherally, in the superficial laminae of the spinal cord and several brain stem nuclei. This suggests that clonidine's analgesic effects are more pronounced alter neuraxial administration^10^.

Clonidine synchronously decreases the cold-response threshold while slightly increasing the sweating threshold^11^,^12^ thus suggesting that it acts on the central thermoregulatory system rather than preventing shivering peripherally^13^.

### Adverse effects:

Administration of clonidine may be accompanied by drowsiness, dry mouth, bradycardia, orthostatic hypotension and impotence. Abrupt withdrawal of the drug could lead to rebound hypertension resulting in a hypertensive crisis. Hence clonidine should be continued throughout the perioperative period. Clonidine may increase blood glucose concentration by inhibiting insulin release^14^.

### Drug interactions

Tricyclic antidepressant drugs and presumably phenothiazines and butyrophenones interfere with the action of clonidine. Although administration of a butyrophenone (e.g droperidol) to a patient taking clonidine, guanabenz, or guanfacine chronically could theoretically precipitate a hypertensive crisis, none has been reported. Acute clonidine or dexmedetomidine administration decreases anaesthetic requirements by 40% to 60% and chronic administration decreases requirements by 10% to 20%^15^–^17^.

### Available forms:

Clonidine is available as tablets, injections and transdermal patches. The various routes and doses are given in Table I.

**Table:** 

Route	Dose
Intranasal	2-4 mcg/kg^18^,^19^.
Intramuscular	2 mcg/kg^20^.
Oral	4-5 mcg/kg^19^,^21^,^22^.
Rectal	2.5-5 mcg/kg with atropine 40 mcg/kg^23^,^24^.
Intravenous	1-2 mcg/kg as a bolusq^13^. 0.18-3.16 mcg/kg/hour^25^. 1 mcg/kg/hour with midazolam 50mcg/kg/hour as an infusion^26^.
Caudal anaesthetic adjuvant	1-2 mcg/kg^10^.
Spinal anaesthetic adjuvant	1-2 mcg/kg^10^.
Epidural anaesthetic adjuvant	0.0625% Bupivacaine with fentanyl 1 mcg/ml and clonidine 0.6 mcg/ml at a rate of 0.2 ml/kg/hour^27^.
Sciatic block	0.2% ropivacaine 0.4 mg/kg/hour with clonidine 0.12 mcg/kg/hour infusion^28^.
Paravertebral block	Bolus of 0.5% bupivacaine 19 ml with clonidine 150 mcg/kg given every 48 hours for 3 weeks via an indwelling catheter^29^.

### Antagonist:

The adverse clinical effects of clonidine and dexmedetomidine can be readily reversed with the specific antagonist atipamezole^30^.

### Clinical applications:

#### As a premedicant:

Clonidine in doses of 4 mcg.kg^−1^ orally or intranasally and in doses of 5 mcg.kg^−1^ rectally provides adequate sedation. Routine atropine administration along with clonidine negates the adverse effects like bradycardia and hypotension. In a study by Almenrader et al, it was observed that the onset of sedation was much faster with midazolam (30+/-13/min) as compared to clonidine (38.5+/-14.6 min) but the quality of sedation, acceptance of steal induction and parental satisfaction were better with clonidine than midazolam^19^,^21^. Clonidine has been proven to resolve agitation and hallucination produced by midazolam^18^.

The quality ofsedation produced by alpha-2 agonists differs from sedation produced by drugs that act on GABA receptors^31^ such as midazolam. Clonidine produces sedation by decreasing the sympathetic nervous system activity and the level of arousal. The result is a calm patient who can be easily aroused to full consciousness. Drugs that activate GABA receptors produce a clouding of consciousness and can cause paradoxical agitation as well as tolerance or dependence.

Clonidine is devoid of respiratory depressant action and lacks the negative effects on cognition, memory and behaviour as seen with midazolam. Thus it may be substituted for premedication^32^,^33^. The taste of clonidine is much better than midazolam^21^. Intranasal administration produces nasal burning^18^ and it offers no advantage over the oral route. It is also reported that the onset of action is faster with the oral administration than with intranasal administration^19^. Oral clonidine with atropine can also be recommended to sedate outpatients^34^.

Jatti et al concluded that clonidine produces good sedation and causes less effect on psychomotor functions and therefore it can be used as a premedicant in children^34^. Oral clonidine attenuated the hyperglycemic response, probably by inhibiting the surgical stress-induced release of catecholamines and cortisol^35^. In doses of 4 mcg/kg oral clonidine blunted the increase in heart rate after intravenous atropine in awake children, although clonidine 2 mcg/kg did not. A larger dose of atropine was required to increase the heart rate by 20 beats/min in children who had received 4 mcg/kg of clonidine^36^. It does not affect the preoperative gastric fluid pH and volume in children^37^.

Clonidine decreases postoperative oxygen consumption and adrenergic stress response. Despite dose dependent adverse effects such as hypotension, sedation and idiosyncratic adverse effects such as bradycardia, clonidine does not induce profound respiratory depression. It mildly potentiates opiate-induced respiratory depression^38^–^41^. Rectal premedication with clonidine was associated with a significant reduction of pain in the early postoperative period as compared to midazolam and was also associated with moderately increased sedation during the first 24 hours postoperatively. The sedative effect of clonidine is in agreement with the unambiguous finding of a parental preference for a calm and sedated child during the first 24 hours postoperatively^23^. Shiga et al observed that oral clonidine premedication does not alter the efficacy of a simulated epidural test dose containing epinephrine or isoproterenol^42^.

### As an adjuvant to regional techniques:

Physiology in newborn and infants differ from older children and adults because of their narrow therapeutic window and increased incidence of toxicity. Some of the documented complications of caudal are by the local anaesthetics and/or their additives. Inadvertent intravasation of bupivacaine has serious CVS and CNS toxicity. Enantiomers like ropivacaine and levobupivacaine are safer and their duration of action can be prolonged by adjuvants like clonidine and ketamine^43^–^46^. The incidence of side effects are lower with clonidine as an adjuvant when compared to morphine given epidurally^47^,^48^. Epidural bupivaeaine with clonidine as a patient controlled epidural analgesia in children and adolescents following extensive spinal surgery should be encouraged due to the low incidence of side effects like postoperative nausea and vomiting (PONV)^27^. Addition of clonidine or ketamine for continuous epidural infusion of ropivacaine following lower limb surgeries provides adequate analgesia. It also enables early diagnosis of compartmental syndrome, as the increase in requirement of analgesics precede other clinical symptoms by an average of 7.3 hours^49^.

A continuous infusion of 0.2% ropivacaine 0.4 mg/kg/hour with clonidine 0.12 mcg/kg/hour through a sciatic nerve catheter offered complete pain relief in a three year old boy who had a subtotal amputation of his foot^28^. In a case of herpetic neuralgia refractory to medical therapy, paravertebral nerve block with a catheter inserted at T2-3 level using 19 nil of 0.5% bupivacaine with 150 mcg of clonidine every 48 hours for three weeks in a pediatric intensive care unit was helpful^29^. A combination of S(+) enantiomer of ketamine 1 mg/kg with clonidine 1 mcg/kg administered caudally is adequate for subumbilical surgery without adverse effects^50^.

Subarachnoid block with bupivacaine and clonidine in term and former preterm infants caused episodes of bradycardia and apnea without desaturation for the first 24 hours postoperatively which resolved spontaneously^51^. Unlike spinal opioids, clonidine does not cause Urinary retention and may hasten the time to first micturition after spinal anaesthesia^14^,^52^–^54^. At the doses of 1-2 mcg/kg, clonidine significantly increases (approximately by a factor of two) the duration of blockade with no haemodynamic effects and decreases the peak plasma concentration of the local anaesthetics.

Caudal 0.2% ropivacaine 0.75 ml/kg with clonidine 1 mcg/kg for subumbilical surgery attenuates changes in postoperative cortisol, insulin and blood glucose response to surgery^55^. The addition of clonidine 2 mcg/kg to a weak (0.2%) solution of ropivacaine could enhance analgesia but reduce the risk of motor blockade^56^. Sharpe P et al in their study concluded that there was an increase in analgesic duration with increasing doses of clonidine administered caudally and arousal time was also prolonged^57^. Light to moderate sedation is commonly observed postoperatively for 1 to 3 hours, which is more beneficial than detrimental in paediatric patients, and at doses not exceeding 2 mcg/kg, this sedation does not preclude hospital discharge. Using clonidine makes catheter placement unnecessary for many paediatric procedures, reducing the overall morbidity and cost of the regional block procedure. However, there are some respiratory concerns about very young patients especially the premature inftants^58^.

### Analgesic adjuvant:

After systemic administration, clonidine improves the analgesic effects of anti-inflammatory agents and has peripheral (intra-articular, intravenous, regional) antinociceptive effects in combination with local anaesthetics, opioids and ketamine^59^. It is an effective analgesic and sedative in combination with NSAIDS for ophthalmic surgery^60^, tonsillectomy and adenoidectomy^61^. The analgesic effect of clonidine 2 mcg/kg as an adjuvant to 0.25% bupivacaine is similar when administered intravenously or caudally^62^.

### Prevention of emergence agitation:

In a study by Schmidt et al, premedicating children with oral midazolam 0.5 mg/kg or clonidine 4 mcg/kg or transmucosal dexmedetomidine 1 mcg/kg produced the same level of anxiety and sedation postoperatively, but children who were given clonidine or dexmedetomidine had less perioperative sympathetic stimulation and postoperative pain as compared to children who were given midazolam^22^. Children who received intravenous clonidine 2 mcg/kg following induction of teneral anaesthesia woke up slowly (22' vs 14') had a longer PACU stay (57' vs 46') and were sleepy after discharge (75% vs 39%) (p < 0.03) as compared to the placebo group^63^.

### Decreasing Minimum Alveolar Concentration (MAC) of sevroflurane:

Nishina et al in their study found that oral clonidine 4 mcg/kg given 105 minutes before induction decreased MAC values of sevoflurane for LMA insertion. The MAC of sevoflurane in the clonidine group was 1.3% +/- 0.18% and in the placebo group it was 2% +/- 0.1 6^64^. The combination of clonidine and nitrous oxide lessened the MAC of sevoflurane more than that achieved by either drug alone^65^.

### Postoperative nausea and vomiting (PONV):

Handa et al has shown that premedication with 4mcg/kg of oral clonidine 105 minutes before paediatric strabismus surgery enhances the antiemetic effect of propofol when compared with oral midazolam 0.4 mg/kg^66^. Both oral and caudal clonidine has been reported to reduce the incidence of postoperative vomiting in children^67^–^69^.

### Controlled hypotension:

In adolescents aged 10 – 16 years, oral clonidine 5 mcg/kg on the night before surgery and 90 minutes before a major oromaxillofacial surgery reduced the dose of anaesthetics, analgesics, hypotensive agents and provided faster recovery from anaesthesia. It also reduced the fluctuations in blood pressure and heart rate perioperatively^70^.

### Sevoflurane induced agitation:

Bock et al found that prophylactic use of clonidine decreased sevoflurane induced agitation at a dose of 4 mcg/kg, independent of the route of administration^71^ without increasing postoperative side effects in children^72^.

### In cardiovascular surgery:

Intravenous clonidine 0.18 to 3.16 mcg/kg/hr was found to be an effective analgesic, sedative and it ensured haemodynamic stability by decreasing withdrawal symptoms like CNS hyperactivation, hypertension, tachycardia and fever following surgery to correct congenital heart defects in infants aged 0–24 months. There was an age related normalized profile of the haemodynamic parameters with a reduction in heart rate and mean arterial pressure from the upper norm to the mean within 24 hours. In no case, was there a fall in blood pressure which required additional therapy to reach the target blood pressure^25^.

### Post operative shivering:

Clonidine is effective in treating post operative shivering in children^73^. In a study by Bergendahl et al^23^, clonidine prevented postoperative shivering when compared to midazolam. Extrapolation from adult data revealed that a dose of 1.5 mcg/kg is required to stop shivering in 5 minutes alter drug administration^74^.

### Daycare Surgery:

Oral clonidine premedication and new safer local anaesthetics like ropivacaine and levobupivacaine with adjunvants like clonidine or ketamine for reaional blocks and single caudal shots prolong analgesia with minimal side effects. These have been useful adjuncts in pediatric ambulatory surgery. Behavioural and cognitive changes may be seen. Hence parental education prior to administration is important^75^,^76^.

### Attenuation of response to tracheal intubation and extubation:

It was found that children premedicated with rectal clonidine 2.5 mcg/kg did not have a rise in neuropeptide Y, a marker of major adrenergic activation during tracheal intubation, compared to those who received nmidazolam 300 mcg/kg^24^. It was also found that oral clonidine 4 mcg/kg given 105 minutes before induction attenuated hemodynamic changes associated with tracheal extubation. Yabuchi et al in their study found that oral clonidine premedication decreased MAC of sevoflurane for tracheal extubation and did not prolong emergence from anaesthesia^77^.

### Anaesthetic sparing effect:

Oral clonidine premedication in children at a dose of 2-4 mcg/kg decreases the dose of intravenous barbiturate required for induction of anaesthesia and also reduces halothane requirement for maintenance of anaesthesia^17^,^78^.

### Treatment of spasticity:

Baclofen and clonidine are used in children diagnosed with cerebral palsy or traumatic brain injury. Mean dosages of 40 mg/day (n=86) and 0.4 mg/day (n=31) were required for baclofen and clonidine, respectively. The maximum dosage was 240 mg/day for baclofen and 3.8 mcg/kg for clonidine^79^.

### Ventilatory response:

Clonidine administered caudally in a dose of 1 mcg/kg did not produce a rise in EtCO_2_, despite prolonged sedation. Nishina et al found that a premedicant dose of 4 mcg/kg oral clonidine did not attenuate the increase in minute volume induced by a hypercapneic challenge under sevoflurane anaesthesia. They found no difference in the respiratory rate, EtCO_2_ and SpO_2_ between clonidine and placebo groups and suggested that clonidine is a suitable premedicant for children to undergo sevoflurane anaesthesia with spontaneous ventilation^80^. Infants who were preterm, formerly preterm or in the neonatal period had perioperative apnea following caudal clonidine^81^–^83^

### Cyclical vomiting syndrome:

Palmer et al reported that intravenous clonidine found relief in a teenage boy with cyclical vomiting syndrome not responding to conventional therapy. He suggested that there are links between migraine, cardiovascular system and adrenergic autonomic dysfunction^84^.

### Sensorymotor gating deficits:

Clonidine because ofits effect on alpha-2c receptors is used to treat sensorymotor gating deficits like attention deficit hyperactivity disorder ADHD^85^ schizophrenia, post traumatic stress disorder and drug withdrawal^86^.

### Sedation in Paediatric Intensive Care Unit (PICU):

Clonidine is used as an analgesic and sedative in the ICU^87^ and forms a part of the ICU protocol in UK^88^. Intravenous clonidine 1 mcg/kg/hour with midazolam 50 mcg/kg/hour was not associated with significant changes in heart rate, blood pressure and cardiac index and achieved satisfactory sedation scores^26^. Hence clonidine was found to be cardiostable as a sedative along with midazolam in critically ill infants who were ventilated^26^,^89^. Lowery et al, has reported a long term use of about four and a half months in a critically ill infant for analgosedative purpose^89^. Lyons et al reported a case of an 11 year old child with 78% deep burns who was ventilator dependent due to the use of large doses of morphine. Addition of low dose clonidine to the analgesic regime produced a dramatic reduction in morphine consumption with an attendant improvement in ventilatory, gastrointestinal and psychological functions^90^.

### Clonidine Overdose:

Caudal clonidine has a large margin of safety in healthy children as reported in three cases where 100 times the dose for a single shot caudal was given. Apart from excessive somnolence for a day, these children had no respiratory depression or haemodynamic instability^91^. A five year old child with cerebral palsy and seizure disorder was given clonidine in excessive doses by the mother to control restlessness. The child had bradycardia and hypotension after induction and required resuscitation^92^. In a multicentre study conducted by Spiller et al children younger than twelve years of age who reported to six poison centers with clonidine ingestion were followed for a minimum of 24 hours. Though clinical effects were common, severe adverse effects occurred only in 10% of the patients. The dose ingested was reported for 90 patients (80%). 61 (68%) children ingested 0.3 mg and none had coma, respiratory depression, or hypotension. The lowest dose ingested that resulted in coma and respiratory depression was 0.3 mg (0.015mcg/kg). The authors have recommended a direct medical evaluation for (1) all children 4 years of age and younger with unintentional clonidine ingestion of 0.1 mg (2) ingestion of 0.2 mg in children 5 to 8 years of age and (3) ingestion of 0.4 mg in children older than 8 years of age. Observation for 4 hours may be sufficient to detect patients who will develop severe effects^93^.

Sinha et al reviewed cases of clonidine poisoning presenting to Royal Children's Hospital, Melbourne, Australia over the period from 1997 to 2001. Twenty-four cases of clonidine poisoning were identified over the 5 year period. Nine patients ingested their own medication, which was prescribed for attention-deficit hyperactivity disorder. Clonidine was prescribed for children in 16 cases (67%) for other purposes. Impaired conscious state and bradycardia were the most common presenting features. Activated charcoal was given in 14 cases and volume expansion in six. There were 12 children (50%) who required admission to the intensive care for monitoring, including three who received mechanical ventilation. The average length of stay was 25.7 hours with no long-term complications^85^.

### Contraindications to the use of Clonidine:

Hypovolemia, A-V block, prolonged P-R interval and spontaneous bradycardia^94^.

## Conclusion:

Clonidine is associated with a number of beneficial effects especially in the paediatric age group. Its ability to provide a calm patient preoperatively, stable intraoperative haemodynamics and a prolonged post-operative sedation without respiratory depression makes it a suitable anaesthetic agent. Furthermore, the analgesic effect provided by clonidine when administered intravenously and as an adjuvant to regional anaesthetic techniques makes it a suitable choice in infants. Because of its sedative, anxiolytic and analgesic properties, clonidine is assuming greater importance as an anaesthetic adjuvant in paediatric anaesthesia.
